# ERCP and laparoscopic cholecystectomy in a combined (one-step) procedure with a novel technique

**DOI:** 10.1186/1471-2482-13-S1-A15

**Published:** 2013-09-16

**Authors:** Luigi De Santis

**Affiliations:** 1 U.O.C of Surgery Piove di Sacco (Padova), Italy

## Background

This study compared the benefit of the novel one- step procedure for the managment of calculous biliary disease.

## Methods

A retrospective review of 25 geriatric patients was conducted one-step procedure with positionament of loop in digiuno for reductions a little bowel distenction. In 24 of the patients, the one-step technique was successful (96 %); in the remaining 1 patient (4%), conversion open technique was necessary; in this case not positionament of loop in jejunum. We define the one-step procedure to be a laparoscopic cholecystectomy whit IOC to confirm the presence of stone, to be a gold standard; intraoperative ERCP whit stone extraction was conected if necessary as part of the one-step procedure.

## Result

This technique evidence a statistically significant difference of the hospital cost, such us the length of the stay and pre operative day and reduce the high conversion rate with alternatively technique in two-step. The incidence of overall complications was lower in the one-step technique. The findings showed that one-step technique was associated with less clinical pancreatitis respect at the two-stage technique.

## Conclusion

A laparoscopic cholecystectomy after ES is lengthier and more difficult then in uncomplicated cholelithiasis and should therefore be performed by an experienced surgeon. This new technique appears to be a significant conversion reduction versus two step procedure, and reduce the cost of hospitality and length of stay and preoperative days. Further research with a larger study population is necessary to determine the additional benefits of this procedure.

## References

[B1] Noel R Enochsson L Swahn F Löhr M Nilsson M Permert J Arnelo U A 10-year study of rendezvous intraoperative endoscopic retrograde cholangiography during cholecystectomy and the risk of post-ERCP pancreatitis Surg Endosc 2013 23355164 10.1007/s00464-012-2768-4

[B2] Reinders JS Gouma DJ Heisterkamp J Tromp E van Ramshorst B Boerma D Laparoscopic cholecystectomy is more difficult after a previous endoscopic retrograde cholangiography HPB (Oxford) 2013 15 3 230 4 10.1111/j.1477-2574.2012.00582.x 23374364 PMC3572285

[B3] Wang B Guo Z Liu Z Wang Y Si Y Zhu Y Jin M Preoperative versus intraoperative endoscopic sphincterotomy in patients with gallbladder and suspected common bile duct stones: system review and meta-analysis Surg Endosc 2013 23355158 10.1007/s00464-012-2757-7

[B4] Jones M Johnson M Samourjian E Slauch K Ozobia N ERCP and laparoscopic cholecystectomy in a combined (one-step) procedure: a random comparison to the standard (two-step) procedure Surg Endosc 2012 23239300 10.1007/s00464-012-2647-z PMC4050060

[B5] Pereira-Graterol F Venales-Barrios Y Bousquet-Suárez J Cáceres-Cauro A Romero-Bravo C Moreno-Rodríguez J Rodríguez-Perero L [The "rendez-vous" maneuver as a technical option to access the bile ducts: Case series report] Rev Gastroenterol Mex 2012 77 4 224 8 10.1016/j.rgmx.2012.04.011 23153415

[B6] Arezzo A Vettoretto N Famiglietti F Moja L Morino M Laparoendoscopic rendezvous reduces perioperative morbidity and risk of pancreatitis Surg Endosc 2013 27 4 1055 60 doi: 10.1007/s00464-012-2562-3 10.1007/s00464-012-2562-3 23052536

